# Fusion of hyperspectral imaging (HSI) and RGB for identification of soybean kernel damages using ShuffleNet with convolutional optimization and cross stage partial architecture

**DOI:** 10.3389/fpls.2022.1098864

**Published:** 2023-01-18

**Authors:** Ling Zheng, Mingyue Zhao, Jinchen Zhu, Linsheng Huang, Jinling Zhao, Dong Liang, Dongyan Zhang

**Affiliations:** National Engineering Research Center for Agro-Ecological Big Data Analysis & Application, Anhui University, Hefei, China

**Keywords:** soybean damages, hyperspectral imaging, super resolution, image fusion, lightweight deep learning

## Abstract

Identification of soybean kernel damages is significant to prevent further disoperation. Hyperspectral imaging (HSI) has shown great potential in cereal kernel identification, but its low spatial resolution leads to external feature infidelity and limits the analysis accuracy. In this study, the fusion of HSI and RGB images and improved ShuffleNet were combined to develop an identification method for soybean kernel damages. First, the HSI-RGB fusion network (HRFN) was designed based on super-resolution and spectral modification modules to process the registered HSI and RGB image pairs and generate super-resolution HSI (SR-HSI) images. ShuffleNet improved with convolution optimization and cross-stage partial architecture (ShuffleNet_COCSP) was used to build classification models with the optimal image set of effective wavelengths (OISEW) of SR-HSI images obtained by support vector machine and ShuffleNet. High-quality fusion of HSI and RGB with the obvious spatial promotion and satisfactory spectral conservation was gained by HRFN. ShuffleNet_COCSP and OISEW obtained the optimal recognition performance of ACC_p_=98.36%, Params=0.805 M, and FLOPs=0.097 G, outperforming other classification methods and other types of images. Overall, the proposed method provides an accurate and reliable identification of soybean kernel damages and would be extended to analysis of other quality indicators of various crop kernels.

## 1 Introduction

Soybean is one of the most important legume crops used as human food and animal feed in the world; it has 18%–22% oil and 38%–56% vegetable protein in its seeds (Arslan et al., 2018). Soybean has a protective effect against many diseases, such as high cholesterol, osteoporosis, cardiovascular, chronic diseases, and cancers (Xiao, 2008). The shell of soybean kernels is easily broken during transportation and storage because of its weak protective morphological arrangement; as such, soybeans are susceptible to mildew in the high-temperature and muggy atmosphere due to the post-maturation effect (Rani et al., 2013; Bessa et al., 2021). In broken and moldy soybean kernels, proteins and lipids undergo degradation more readily during storage, leading to quality deterioration (Yousif, 2014). Identification of damaged soybean kernels is prerequisite and conducive to reduce the infection of healthy kernels to ensure the quality of subsequent product and avoid economic loss.

Commonly used methods for soybean damage detection include morphological analysis, chemical analysis, and imaging techniques (Zhao et al., 2011; Yang et al., 2015; Adão et al., 2017). Morphological analysis requires the operator to be experienced and is susceptible to subjective interference. Chemical analysis, such as chromatography and enzyme-linked immunosorbent assay, owns high accuracy and excellent reproducibility but is a destructive, time-consuming, and labor-intensive process. Imaging techniques, such as red–green–blue (RGB) imaging with high spatial resolution and hyperspectral imaging (HSI) with high spectral resolution, have been popularized in image classification, object detection, and semantic segmentation. However, subtle changes in the internal composition of the kernels are difficult to be perceived by RGB due to insufficient spectral information (Steinbrener et al., 2019).

HSI can simultaneously provide spectral responses and spatial images of hundreds of continuous wavelengths to obtain spectral and external features, thereby enriching the description of soybean kernels (Lu et al., 2020). HSI hardware typically sacrifices spatial resolution to ensure premium spectral resolution due to limited incident energy (Dian et al., 2021). The low spatial resolution leads to weak fidelity of appearance-based features especially when discriminating small objects, such as soybean kernels (Fabiyi et al., 2020). This problem can be solved by multi-modal image fusion, which extracts and combines the most meaningful information from images of different sources to generate a single image that is more informative and beneficial for subsequent applications (Zhang et al., 2021). Thus far, the methods for fusing HSI and RGB images can be broadly divided into multi-scale transformation based on coefficients (Wei et al., 2021), saliency (Muddamsetty et al., 2013), sparse representation (Wei et al., 2015), and deep learning (Wei et al., 2017). Fusion rules in the first three categories are specifically designed in the transform or spatial domain in virtue of transform bases. However, applying the same transformation basis such as wavelet basis (Starck et al., 2001) and ridgelet basis (Chen et al., 2005) to HSI and RGB images may lead to confined fusion performance (Yang and Li, 2012).

In recent years, deep learning-based fusion methods can extract diverse and multi-scale features to achieve adaptive fusion. Notably, the absence of ground truth (GT) images in real scenes leads to the inapplicability of widely used supervised learning models (Zhang et al., 2021). Therefore, an unsupervised method has practical significance (Wang et al., 2020). Proposed an unsupervised and coupled autoencoder (AE) framework implemented by CNNs for super-resolution HSI. However, continuous convolution leads to the loss of information from shallow layers containing low-level features at high spatial resolution, which is unbeneficial for fusion. Dense connections enhance feature propagation and improve information flow by interconnect layers and bypass settings, thereby providing continuous attention of features and preserving the detailed information of HSI and RGB images (Dolz et al., 2018).

Spectral preservation plays an crucial role in fusing HSI and RGB images due to the skewed spectral information that affects the quality of the fused image (Hu et al., 2021). Channel attention is commonly used to assign feature importance by dynamically adjusting the weight of each channel to assist the performance improvement of various task; they can also be used to correct the spectral information in image fusion (Hu et al., 2017).

Although a number of approaches are available for constructing super-resolution hyperspectral image (SR-HSI), few researchers focus on the identification effect of SR-HSI in real environment. End-to-end neural networks use translation invariance and rotation invariance to automatically extract key features without manual feature engineering in image recognition applications (Dhaka et al., 2021; Kundu et al., 2021). However, the high computing and memory requirements hinder the application of complex networks. Lightweight networks, such as MobileNetV2 (Sandler et al., 2018), GhostNet (Han et al., 2020), and ShuffleNet (Ma et al., 2018), which have small parameter and low computation, can achieve good accuracy on resource-constrained devices. In particular, the efficient architecture of ShuffleNet solves the boundary effect problem caused by depth-wise (DW) separable convolution. Convolution optimization including pruning the redundant convolution layer and enlarging the convolution kernel can accelerate the network inference speed and extract richer global features (Luo et al., 2016). The CSP architecture with switching concatenation and transition steps as shortcut operation allows the gradient flow to propagate through different paths of network to enrich the gradient combination and quicken the rate of reasoning (Wang et al., 2020). ShuffleNet can be combined with convolution optimization (CO) and CSP architecture to identify damages to soybean kernels.

**Figure 1:**
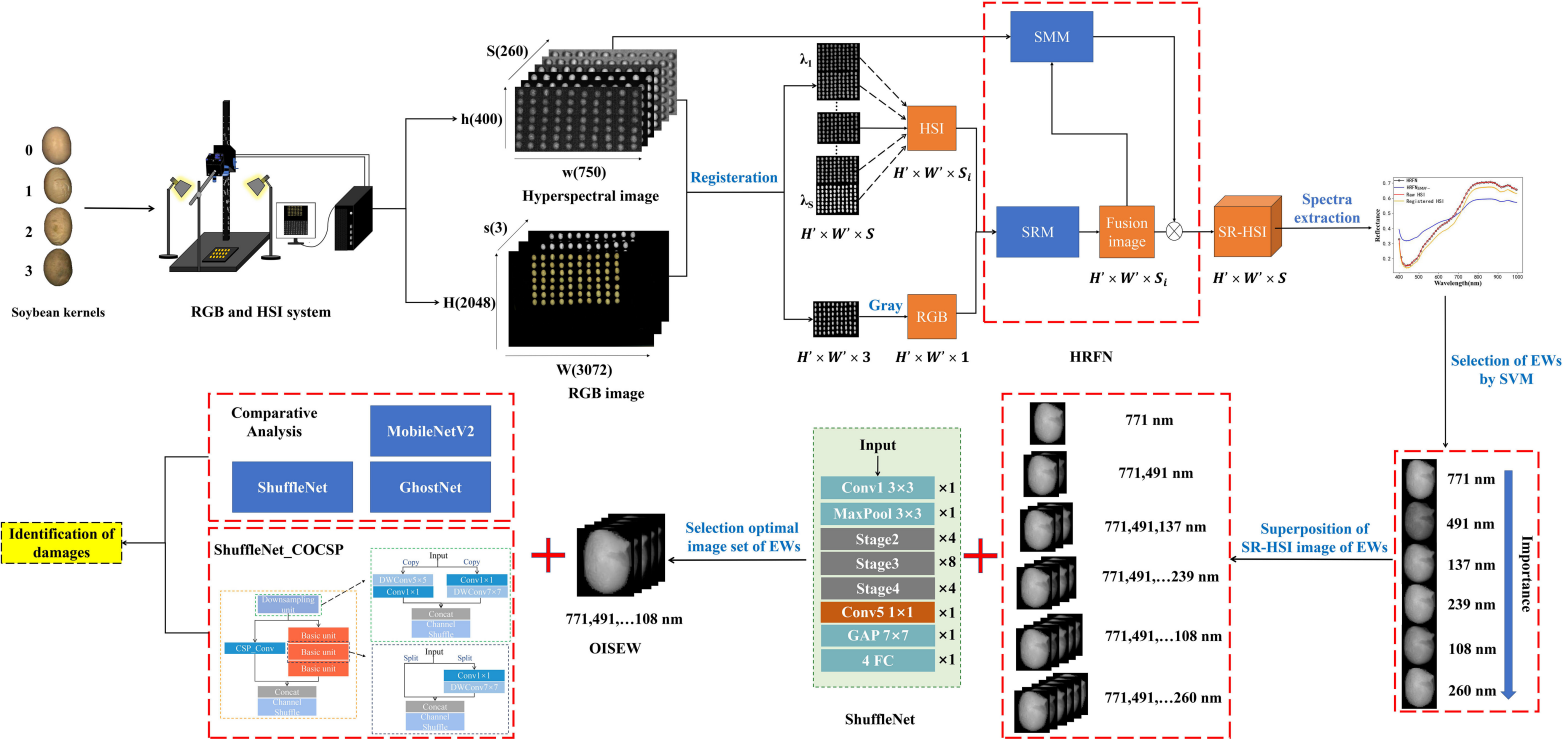
Flowchart of identification of soybean kernel damages; Class 0-3: healthy, broken, mildly moldy and severely moldy soybean kernels; SRM is designed by Autoencoder and Dense block.

Herein, fusion of HSI and RGB images and improved ShuffleNet were proposed to identify soybean kernel damages (**Figure 1**). First, a super-resolution module based on AE and dense connection and spectroscopy-modification module from the idea of channel attention were designed and integrated to construct a HSI-RGB fusion network (HRFN) and generate SR-HSI images. SVM and ShuffleNet were used to select the SR-HSI monochromatic images of effective wavelengths for rapid identification of soybean kernel damages. Finally, a new identification network architecture, namely, ShuffleNet _COCSP, was developed by combining CO and CSP architectures with ShuffleNet to identify soybean damages with SR-HSI. The main contribution of this study can be summarized as follows.

1. To the best of our knowledge, this study is the first to fuse the HSI and RGB images of small kernels and develop the lightweight network ShuffleNet _COCSP for practical identification of damages.
2. The proposed novel network for HSI and RGB image fusion consists of parallel super-resolution module (SRM) and spectral correction module (SMM).
3. An improved efficient ShuffleNet with convolution optimization and cross-stage partial is proposed for accurate identification of soybean kernel damages.

## 2 Materials and methods

### 2.1 Sample preparation

Samples of He13 soybean kernels (**Figure S1** in **Supplementary Material**) were obtained from Shu County Agricultural Management Company. Soybeans with smooth surface were selected and considered as healthy ones. Healthy soybeans were soaked in warm water for 10 min and dried using a drying oven at 100 °C for 2 h to obtain broken soybeans. Moldy soybeans were prepared as follows. 1) Healthy soybeans were soaked in warm water for 10 min and placed in a glass Petri dish. 2) The dish was placed in an incubator with constant temperature of 34°C and humidity of 80% to obtain different degrees of moldy soybeans. 3) Moldy soybeans were collected daily. Mildly moldy soybeans have few spots on the epidermis, whereas severely moldy soybeans have mycelia on the epidermis.

Aflatoxin B1 test strip was used to determine the toxin of moldy soybeans. The lower limit of AFB1 toxin detection was 10 ppb, mildly moldy was in the range of 10–20 ppb, and severely moldy was greater than 20 ppb. For HSI and RGB image acquisition, 2,160 samples were collected, including 560 healthy kernels, 560 broken kernels, 560 mildly moldy kernels, and 480 severely moldy kernels.

### 2.2 Acquisition and calibration of HSI and RGB images

The image acquisition system of soybean kernels was composed of high-resolution RGB camera and HSI (**Figure S2** in **Supplementary Material**). The industrial camera HIKVISION MV-CA060-11GM with a 12 mm/F2.0 lens was used to collect RGB images at 3072×2048 pixels and save them in BMP format. HSI images were obtained by a visible/NIR HSI system consisting of a Headwall Nano-Hyperspec (Headwall Photonics Inc., Bolton, MA, USA) push-broom sensor that offers 272 spectral bands, two halogen neodymium lamps (75 W), and a computing unit. For imaging, 70 soybean kernels were placed on a black plate, and the distance between the kernels and lens of the HSI sensor and RGB camera was adjusted to 40 cm. Two halogen lamps were placed on both sides of the lens for illumination. During data acquisition, the RGB industrial camera was set to operate in manual mode with an ISO of 400 and a shutter speed of 16 ms. The parameters of the HSI system were set as follows: exposure time, 70 ms; frame period, 70 ms; and scanning speed, 0.45 deg/s. For calibrating the image, white and dark reference images were acquired by scanning a standard white board with 98% reflectance and covering the lens before collecting HSI images. The correction formula is as follows:


(1) $$I_{\text{c}}=\frac{I_{\text{r}}{-I}_{\text{d}}}{I_{\text{w}}{-I}_{\text{d}}}$$

where *I*_c_ is the corrected image, *I*_r_is a measured raw image of soybean kernels, and *I*_w_and *I*_d_ are the white and dark reference images, respectively.

### 2.3 Image preprocessing

The spatial misalignment of source images was caused by the difference between image sensors. In fusion tasks, operations along the spatial pixel positions in deep learning methods are unavailable for real source images due to spatial dislocation (Jiang et al., 2021). As a result, high-precision registration is a key issue in image fusion for constructing SR-HSI datasets. Transformation, rotation, and translation parameters were obtained by perspective deformation to align HSI and RGB images (Arsigny et al., 2005). Specifically, three band images were extracted from the HSI image to form a pseudo-RGB image, which was used as the image to be aligned with the RGB image as the reference image. The region of interest (ROI, rectangle) is selected from the pseudo-RGB image for perspective deformation. Transformation, rotation, and translation parameters were accurately calculated from the ROI vertices. The HSI image was transformed using these parameters to align with the RGB image. The designed registration visualization formula is as follows:


(2) $${\text{ h}}_{1}\left(\text{x},\text{y}\right)=\sum_{\text{i}=1}^{\text{R}}{\text{α}}_{\text{i}}\left(\text{x},\text{y}\right)$$

(3) $${\text{h}}_{2}\left(\text{x},\text{y}\right)=\sum_{\text{i}=1}^{\text{r}}{\text{β}}_{\text{i}}\left(\text{x},\text{y}\right)$$

(4) $$\text{f}\left(\text{x},\text{y}\right)=\frac{{\text{e}}^{{\text{h}}_{2}\left(\text{x},\text{y}\right)}}{{\text{e}}^{{\text{h}}_{1}\left(\text{x},\text{y}\right)}+{\text{e}}^{{\text{h}}_{2}\left(\text{x},\text{y}\right)}}$$

where f(x,y) is the registration visualization map, x and y are pixel coordinates, is HSI image, is RGB image, and R and r are the spectral band number of HSI and RGB images, respectively.

Canny operator detects the contour of kernels in RGB. The Otsu’s algorithm was used in threshold segmentation to obtain a binary image. The background noise of the RGB image was removed, and the mask obtained from RGB segmentation was transformed to HSI space to remove the background noise of the HSI image. Using the same mask, the samples of HSI and RGB data sets had one-to-one correspondence in the subsequent recognition, and inconsistent phenomenon of sample division did not exist.

### 2.4 Fusion of HSI and RGB images

**Figure 2:**
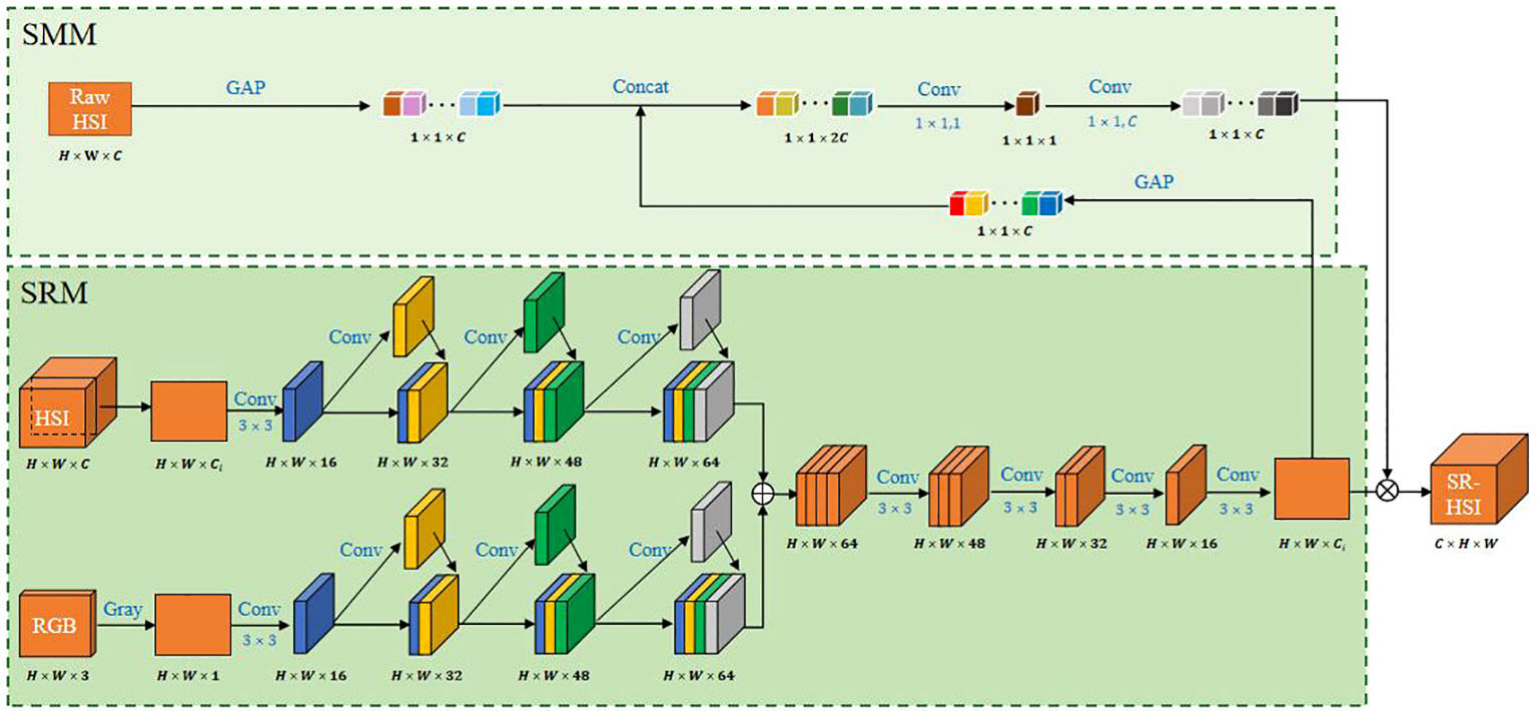
Architecture of HRFN.

In this study, a HSI-RGB fusion network (HRFN) was developed using parallel super-resolution module (SRM) and spectroscopy-modification module (SMM) to solve the problem of low spatial resolution of HSI images. In HRFN (**Figure 2**), the RGB grayscale image and the monochromatic image of 272 bands of HSI were fused to generate the SR-HSI monochromatic image of corresponding band.

SRM was designed based on AE, a widely-used super-resolution deep learning architecture, and dense block. AE is an unsupervised neural network composed of encoder and decoder and has excellent generalization (Liu et al., 2022). The potential representation of images obtained by encoder has valuable attributes, and the concatenated potential representations of multi-modal images can be reconstructed to a high-quality image by the decoder. For super-resolution in our study, the multi-modal images are the registered HSI and RGB image pair. The encoder is composed of four convolutional layers with the kernel size of 3 × 3 and channel of 16; the decoder contains four convolutional layers with the kernel of 3 × 3. However, the successive convolutions make AE suffer from gradient disappearance and inability to maintain shallow and detailed features, which are critical to obtain excellent super-resolution.

Dense connection is introduced to the encoder of AE. In the encoder, the first convolution is a common convolution, and the last three convolution layers are set as a dense block. Dense block can preserve as much information as possible in encoding by the multi re-utilization of features obtained in the former layers.

The super-resolution operation is performed on the registered image pairs, but the registration may lose some important spectral information. With the idea of attention mechanism, SMM, which is in parallel with SRM, is designed to preserve spectral information of the raw HSI. SMM consists of two global average pooling (GAP) layers and two convolution layers of 1×1. The GAP results of the raw HSI and super-resolution image are cascaded and input to the convolutional layers to obtain the weights that describe the correlation between channels. The super-resolution image in SRM is multiplied by the weights to obtain the final SR-HSI image.

### 2.5 SR-HSI images of effective wavelengths

The direct use of SR-HSI images containing the images of 272 wavelengths to identify damages to soybean kernels would result in low processing efficiency and high hardware and time costs. The Images of EWs have been proved as a feasible approach to alleviate the limitations in the previous works (Weng et al., 2021). The selection of EWs from the reflectance spectra was based on the performance of SVM models that describe the reflectance of wavelengths and classes of soybean kernels. Specifically, the reflectance of each wavelength and class for the soybean kernels were employed to develop classification models by using SVM. The higher the classification accuracy is, the more important the wavelength will be. The first six wavelengths were selected as EWs, and the SR-HSI images of EWs were sequentially overlaid on ShuffleNet to select the most suitable wavelength combination for determination of damages to soybean kernels.

Before the above operations, spectra were acquired by the following steps. The SR-HSI image with removed background noise was converted into a binary image by graying and converting to color space HSV. The ROI of the sample was extracted, and the reflectance values of all pixels within the ROI were averaged as the reflectance spectra of the soybean samples.

### 2.6 Recognition model

A deep network with deep architectures possesses powerful feature extraction capability and generally perform well in image tasks. Nevertheless, the high computing and memory requirements of the network hinder its wide application. One approach to solve the problem is the use of a lightweight network. ShuffleNet, which is a powerful lightweight network, can reduce parameters and computation costs by the operation as channels shuffle in the stage layer (Ma et al., 2018).

Specifically, ShuffleNet is composed of convolutional layers, pooling layers, stage layers, and fully connected layers, where the stage is consists of a downsampling unit and a basic unit. These units include DW convolutional layers and 1×1 convolutional layers. However, ShuffleNet replaces a large number of 1×1 point-wise convolutions with channel shuffle to induce the lack of representation ability and slight loss of accuracy. In the stage architecture, convolutional optimization (CO) and cross-stage partial (CSP) architecture were adopted to alleviate the above challenges in this study. The removal of the last convolutional layer and the substitution of the DW convolution kernel size of 3×3 with 7×7 reduce the model parameters, expand the perceptual field, and obtain rich global features (Ding et al., 2022). By replacing all DW convolution 3×3 with 7×7, the padding needs to be changed from 1 to 3, so the resolution of the output feature map remains the same as the original. The CSP architecture firstly divides the feature maps of the downsampling unit into two parts, make them pass through different paths, and concatenate them together in the end of the stage layer. One part passes through the original path, and the other shortcuts directly to the end of the stage. Through the operation, CSP enables richer gradient sets and reduces computation by splitting gradient streams to propagate through different network paths (Wang et al., 2020).

**Figure 3:**
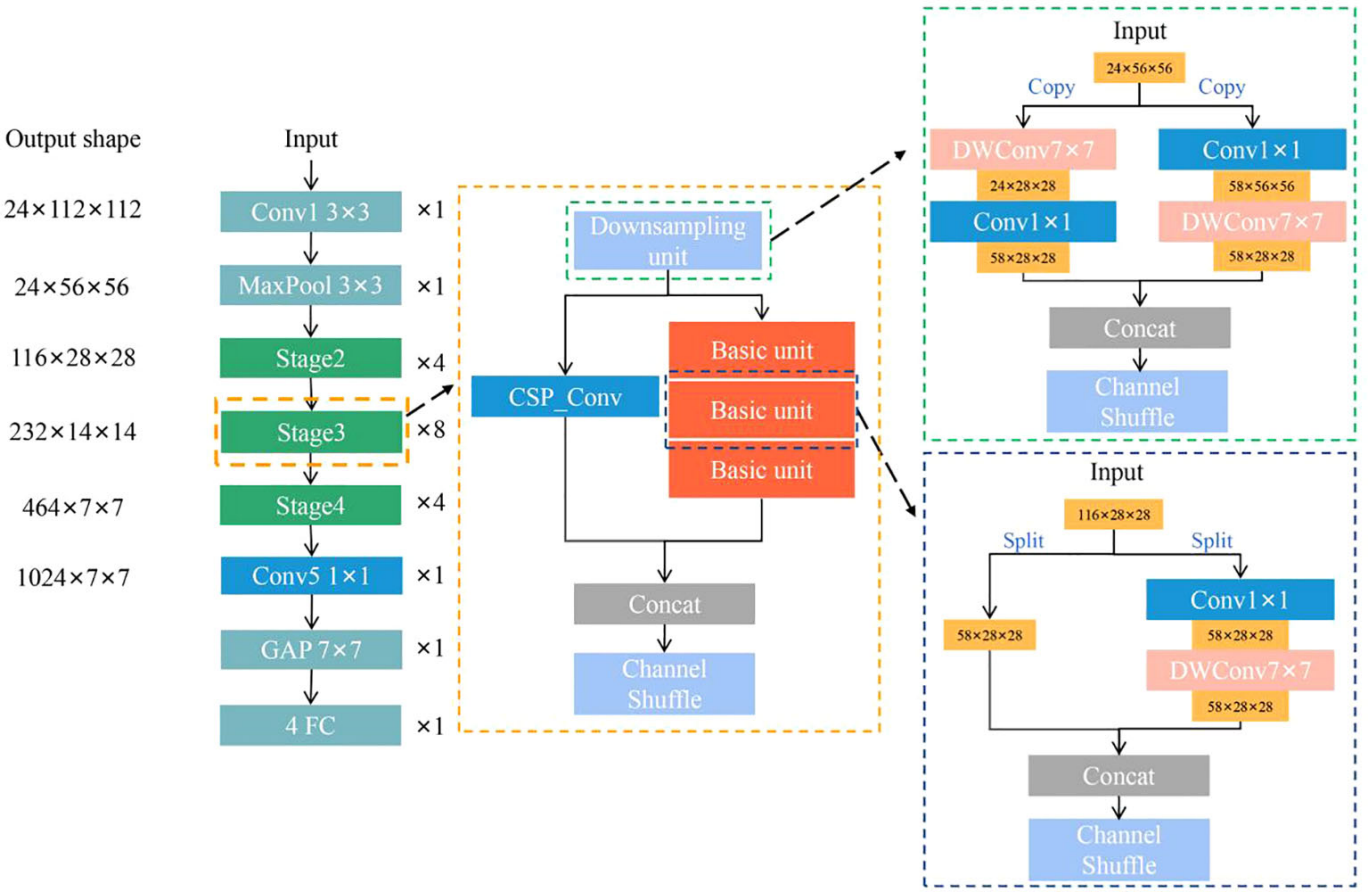
Architecture of ShuffleNet_COCSP.

In this study, ShuffleNet_CO was first constructed by removing convolution and expanding the DW kernel in stage layers based on the ShuffleNet framework. ShuffleNet_COCSP (**Figure 3**) was then developed by introducing the CSP architecture in ShuffleNet_CO. The detailed parameter settings of ShuffleNet_COCSP are shown in **Table S1** in **Supplementary Material**.

### 2.7 Performance evaluation

Mutual information(MI) (Wells et al., 1996), structural similarity (SSIM) (Wang et al., 2004), peak signal to noise ratio (PSNR) (Feng et al., 2012), and mean absolute differences (MAD) (Cheng et al., 2016)are used in registration. The registration performance increases with increasing values of MI, SSIM, and PSNR and decreasing values of MAD, where the ideal values for MI and SSIM are 1. Higher PSNR and lower MAD indicate better quality of registration. Pixel feature mutual information (FMI_pixel_) (Haghighat and Razian, 2014), multi-scale structural similarity (MS-SSIM) (Ma et al., 2015), and Nabf (Xydeas and Petrovic, 2000) are used in fusion. The fusion performance increases with increasing values of FMI_pixel_ and MS-SSIM and decreasing values of Nabf.

Healthy, broken, mildly moldy, and severely moldy soybean kernels were divided into a calibration set, a validation set, and a prediction set according to the ratio of 3:1:1. The calibration and validation sets were used for parameter adjustment and preliminary evaluation of the recognition model. Model performance was quantitatively evaluated using accuracy of calibration set (ACC_C_), validation set (ACC_V_), and prediction set (ACC_P_) as well as precision, recall, and F1-score of the prediction set. The evaluation index of the network was the number of floating point operations (FLOPs) and the number of model parameters (Params). Deep networks were constructed based on the PyTorch framework in Python. All the methods were conducted on a computer with an NVIDIA GeForce RTX 3090 GPU.

## 3 Results and discussion

### 3.1 Image registration

**Figure 4:**
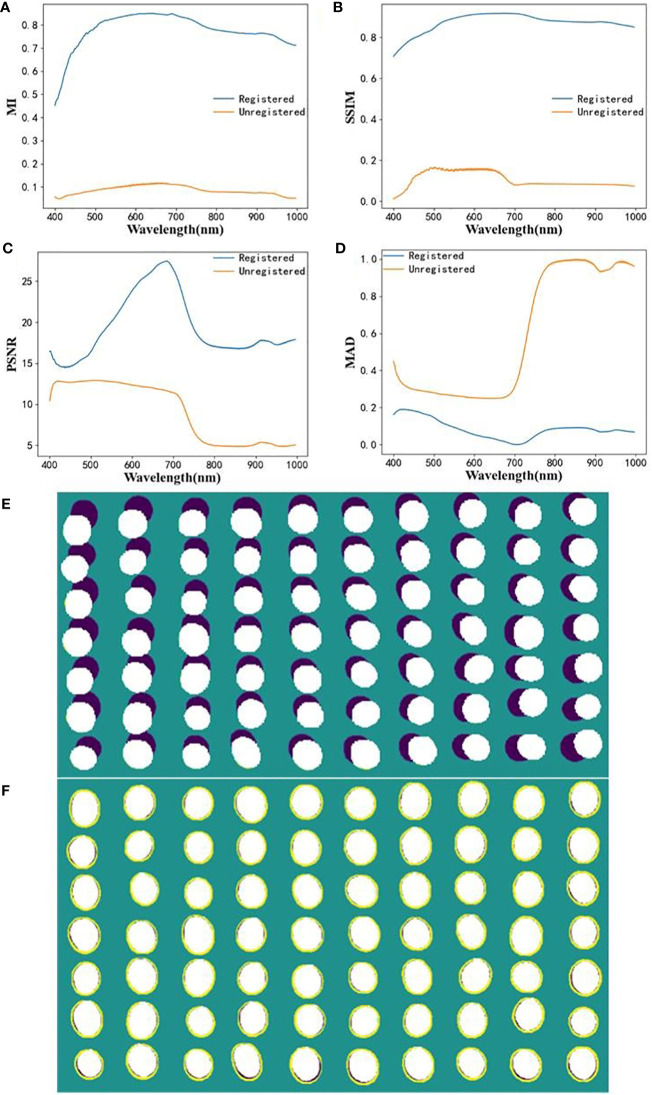
Evaluation results of HSI and RGB registration. **(A)** MI; **(B)** SSIM; **(C)** PSNR; **(D)** MAD; **(E, F)** visualization of image pairs before and after registration.

The registration of HSI and RGB image pairs was performed by perspective deformation, and the performance for healthy soybean kernels is shown in **Figure 4**. From (**Figures 4A, B**), the MI and SSIM of the registered image pairs were higher than 0.46 and 0.71 while those of the unregistered image pairs were lower 0.15 and 0.2. The registration operation greatly improves the structural similarity between HSI and RGB images. The PSNR trend of the registered image pairs increased first and then decreased, while the MAD trend was opposite. From the registration visualization (**Figures 4E, F**), the raw image pairs of HSI and RGB are almost not spatially aligned, but the image pairs are almost perfectly aligned with only minor misalignment at the edge after the registration. Hence, the proposed registration operation satisfactorily solves the spatial dislocation of image pairs.

### 3.2 Fusion of HSI and RGB

**Figure 5:**
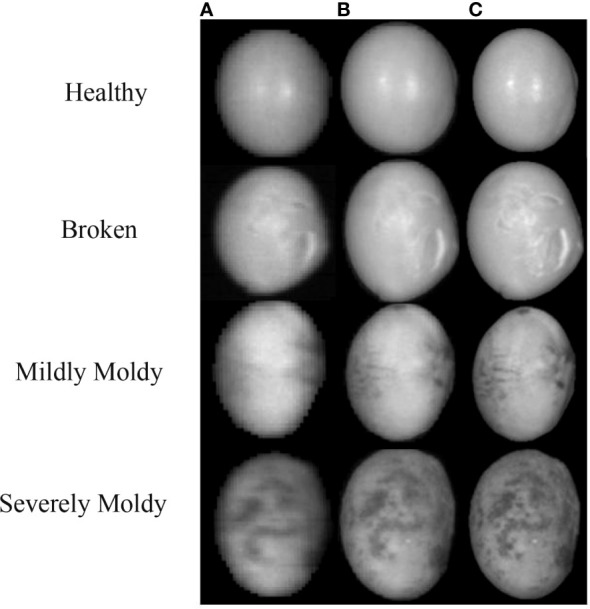
Fused results for HSI and RGB images with HRFN. **(A)** The registered HSI image of the 618 nm, **(B)** SR-HSI image of the 618 nm, **(C)** RGB Gray image.

HRFN was adopted to fuse the registered HSI and RGB pair to generate SR-HSI (**Figure 5**). The texture and color of soybean kernels can be clearly observed in the RGB image (**Figure 5A**), but their details are very blurred in the registered HSI image (**Figure 5B**). SR-HSI has better spatial resolution than HSI (**Figure 5C**) and more spectral bands (272) than RGB (3). In simple terms, SR-HSI can be regarded as the spectral resolution improvement of RGB or the spatial resolution enhancement of HSI. From the quantitative results of soybean kernels in **Table 1**, HRFN was highly effective in the fusion for the four classes of soybean kernels, with the FMI_pixel of 0.9415–0.9614, MS_SSIM of 0.9678–0.9880, and Qabf below 0.0508. The FMI_pixel and MS_SSIM of healthy soybean kernels were 0.9488 and 0.9842 higher than those of the three other classes because of the low values of statistical contrast characteristics of structural information for broken or moldy areas of soybean.

**Table 1:** Fusion performance of HSI and RGB using HRFN.

Method	Classes	FMI_pixel	MS_SSIM	Nabf
HRFN	Healthy	0.9614	0.9880	0.0422
	Broken	0.9437	0.9678	0.0349
	Mildly moldy	0.9494	0.9781	0.0375
	Severely moldy	0.9415	0.9778	0.0508

The reflectance spectra of SR-HSI almost perfectly overlapped with those of raw HSI, indicating that the SMM module learned the mapping relationship between the HSI and super-resolution image (**Figure 5**). Thus, HRFN achieves good fusion of HSI and RGB, and the SMM module retains the spectral information from HSI to improve the quality of SR-HSI.

### 3.3 Selection of image set of EWs

Selecting the key variables of HSI data cube can avoid dimensional disasters and improve the interpretability and generalization ability. Here, the SR-HSI monochromatic images of EWs were extracted, and the optimal image set of EWs (OISEW) was selected according to the classification results of soybean kernel damages. The six EWs selected by SVM are 771, 491, 700, 927, 635, and 973 nm. The SR-HSI monochromatic images of the six EWs were stacked in the above order, and the most remarkable image set was screened based on the performance of ShuffleNet (**Table 2**). The ShuffleNet parameters are presented in **Table S1** in **Supplementary Material**. For the SR-HSI image of 771 nm, poor results were obtained with ACC_T_= 99.12%, ACC_V_= 88.94%, and ACC_P_= 86.97% mainly because of insufficient information of the image of one wavelength. With the addition of images of other wavelengths, the accuracy first increased and then decreased. The best results of ACC_T_= 99.89%, ACC_V_= 95.65%, and ACC_P_=92.27% were obtained using the SR-HSI image sets of 771, 491, 700, 927, and 635 nm, named as OISEW. The classification performance for severely moldy kernels was improved significantly with precision of 86.14%–97.75%, recall of 91.43%–92.55%, and F1-score of 89.23%–95.08%. The results on mildly moldy kernels were similar to those on severely moldy kernels. The classification results of healthy and broken classes were superior to the other image set of EWs, with F1-scores of 90.30% and 90.17%, respectively. Thus, OISEW was adopted to classify the various damages of soybean kernels in subsequent analysis.

**Table 2:** Classification results of soybean kernels using ShuffleNet with SR-HSI images of different combinations of EWs.

SR-HSI image set	Classes	Accuracy (%)	Prediction dataset		
			Precision (%)	Recall (%)	F1-score (%)
771nm	Healthy Broken Mildly moldy Severely moldy	ACC_T_=99.12 ACC_V_=88.94 ACC_P_=86.97	80.81 91.58 89.47 88.14	88.89 87.00 80.19 91.43	84.66 89.23 84.58 89.23
771 and 491 nm	Healthy Broken Mildly moldy Severely moldy	ACC_T_=99.38 ACC_V_=91.54 ACC_P_=90.31	85.57 94.70 90.00 95.56	92.22 89.00 93.40 91.49	88.77 91.53 91.67 93.48
771, 491 and 700 nm	Healthy Broken Mildly moldy Severely moldy	ACC_T_=99.67 ACC_V_=90.84 ACC_P_=90.44	86.46 91.78 96.70 85.85	92.22 89.51 83.02 96.81	89.25 90.37 89.34 91.00
771, 491, 700 and 927nm	Healthy Broken Mildly moldy Severely moldy	ACC_T_=99.86 ACC_V_=94.12 ACC_P_=91.35	86.81 90.82 95.10 91.92	87.78 89.00 91.51 96.81	87.29 89.90 93.27 94.30
771, 491, 700, 927 and 635 nm	Healthy Broken Mildly moldy Severely moldy	ACC_T_=99.89 ACC_V_=95.65 ACC_P_=92.27	87.69 94.44 92.04 97.75	92.22 86.00 98.11 92.55	90.30 90.17 94.98 95.08
771, 491, 700, 927, 635 and 973 nm	Healthy Broken Mildly moldy Severely moldy	ACC_T_=99.82 ACC_V_=94.65 ACC_P_=91.04	80.30 98.85 94.64 94.06	94.64 76.79 94.64 98.96	86.89 86.43 94.64 96.45

### 3.4 Identification of soybean kernel damages using ShuffleNet_COCSP

The ACC_P_=92.27% is insufficient for identification of kernel damages. Thus, ShuffleNet needs to be further optimized considering its efficiency and accuracy. Taking ShuffleNet as backbone, CO and CSP were combined to construct ShuffleNet_COCSP, which was also compared with two widespread lightweight networks, namely, MobileNetV2 and GhostNet. The parameters of each model are shown in **Table S1** in **Supplementary Material**. The identification results of each model are shown in **Table 3**. MobileNetV2 achieved ACC_P_ of 95.95%, Params of 2.231 M, and FLOPs of 0.326 G, and GhostNet achieved ACC_P_ of 95.17%, Params of 4.207 M, and FLOPs of 0.197 G. The identification was satisfactory; however, the point-wise convolutions consume vast and expensive computing resources. ShuffleNet_COCSP obtained the best result with ACC_P_ of 98.36%, Params of 0.805 M, and FLOP of 0.097 G. The ACC_P_ of ShuffleNet_COCSP increased by 5.58%, and the params and FLOPs decreased by 36.01% and 37.42%, respectively, compared with those of ShuffleNet. The F1-scores of ShuffleNet_COCSP for mildly and severely moldy kernels were both 100.00%, and all samples of the two classes were accurately classified. The precision levels of healthy and broken classes were 93.81% and 99.03%, and the recall rates were 98.91% and 94.44%, respectively. Most samples of the 2 classes were identified correctly, with only a small part of broken kernels classified mistakenly as healthy.

**Table 3:** Classification results of soybean kernels using MobileNetV2, GhostNet, ShuffleNet and ShuffleNet_COCSP.

Model	Classes	Accuracy(%)	Prediction dataset			Params (M)	FLOPs (G)
			Precision (%)	Recall (%)	F1-score (%)		
MobileNetV2	Healthy Broken Mildly moldy Severely moldy	ACC_T_=100.00 ACC_V_=97.42 ACC_P_=96.82	93.68 97.17 99.08 96.94	96.74 95.37 96.43 98.96	95.19 96.26 97.74 97.94	2.231	0.326
GhostNet	Healthy Broken Mildly moldy Severely moldy	ACC_T_=99.78 ACC_V_=96.46 ACC_P_=95.37	88.35 100.00 96.46 96.88	98.91 88.89 97.32 96.88	93.33 94.12 96.89 96.88	4.207	0.197
ShuffleNet	Healthy Broken Mildly moldy Severely moldy	ACC_T_=99.88 ACC_V_=94.74 ACC_P_=93.16	86.96 94.06 92.50 98.95	86.96 87.96 99.11 97.92	86.96 90.91 95.69 98.43	1.258	0.155
ShuffleNet_COCSP	Healthy Broken Mildly moldy Severely moldy	ACC_T_= 99.87 ACC_V_= 98.64 ACC_P_= 98.36	95.70 97.20 100.00 100.00	96.74 96.30 100.00 100.00	96.22 96.74 100.00 100.00	0.805	0.097

**Figure 6:**
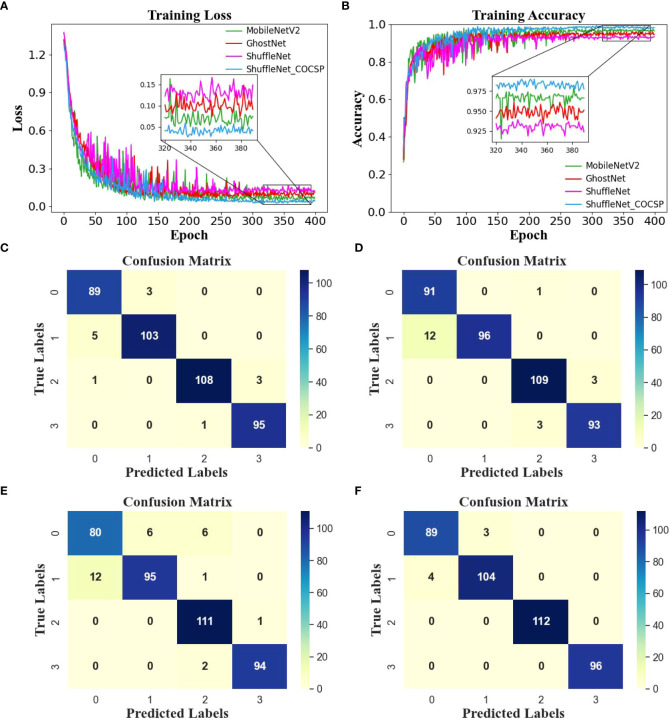
**(A, B)** Learning curves and **(C–F)** confusion matrix of MobileNetV2, GhostNet, ShuffleNet, and ShuffleNet_COCSP in the prediction dataset.

ShuffleNet_COCSP improved the identification accuracy and vastly reduced the computational effort by enlarging the receptive field and removing the redundant convolution layer and CSP shunting techniques. The curves of accuracy and loss (**Figures 6A, B**) showed that ShuffleNet_COCSP was better than MobileNetV2, GhostNet, and ShuffleNet, and the fluctuations of the learning curves gradually decreased. In summary, ShuffleNet_COCSP performed well in the identification of soybean kernel damages with excellent accuracy and efficiency.

## 4 Ablation experiment

In previous studies in the field of fusion of HSI and RGB, the spectral reflectance of a fused super-resolution image was rarely concerned and generally different from that of a raw HSI image. SMM in HRFN was constructed to correct the spectral information and realize good fusion.

HRFN without the SMM module (HRFN_SMM-_) was adopted to fuse the HSI and RGB image pairs and investigate the effect of SMM. The spectral reflectance data of the raw HSI, HRFN, and HRFN_SMM-_ are shown in **Figure 5**. Compared with those of HRFN, the spectra of HRFN_SMM-_ is nonoverlapping with the raw HSI. Thus, SMM can preserve the spectral information of raw HSI because it can learn the mapping relationship between HSI and hyperspectral super-resolution images to obtain the missing spectral information of each band. Based on the fusion results of HRFN_SMM-_ in **Table 4**, its FMI_pixel, MS_SSIM, and Nabf are worse than those of HRFN. As a result, SMM does help HRFN focus on missing spectral details to improve the quality of the SR-HSI image (Hu et al., 2021).

**Table 4:** Quantitative results of fusion HSI and RGB using HRFN_SMM-._.

Method	Classes	FMI_pixel	MS_SSIM	Nabf
HRFN_SMM-_	Healthy	0.9614	0.9865	0.0424
	Broken	0.9435	0.9537	0.0353
	Mildly moldy	0.9491	0.9777	0.0378
	Severely moldy	0.9410	0.9770	0.0509

ShuffleNet_COCSP combined with CO and CSP achieved the ideal identification and was ultra-lightweight. To further corroborate its effectiveness, we employed ShuffleNet with CO (ShuffleNet_CO) and ShuffleNet with CSP (ShuffleNet_CSP) for developing identification models of soybean kernel damages in SR-HSI images (**Table 5**). The ACC_P_ of ShuffleNet_CO and ShuffleNet_CSP increased by 2.79% and 3.46%, the Params decreased by 23.93% and 27.03%, and the FLOPs decreased by 23.26% and 29.68%, respectively, compared with those of ShuffleNet. The results of ShuffleNet_CSP and ShuffleNet_CO were better than that of ShuffleNet and worse than ShuffleNet_COCSP, confirming that CO and CSP played a positive role in recognition. The performance of ShuffleNet_CO is mainly because CO has a large effective receptive field to increase the sensing area of feature maps and extract richer global features. Meanwhile, the redundant 1×1 convolution layer is removed to improve the network efficiency. The Params and FLOPs of ShuffleNet_CSP were greatly reduced because the strategy of truncating the gradient flow was adopted in the CSP architecture; as such, the gradient information will not be reused. Surprisingly, ShuffleNet_CSP has a good accuracy in identifying soybean kernel damages. The reason may be that CSP architecture enhance the variability of the learned features within different layers, thereby greatly improving the learning ability of the network. The advantages of CO and CSP are perfectly combined to make ShuffleNet more efficient and ensure the accuracy of recognition.

**Table 5:** Recognition results based on SHUFFLENET adding CO and CSP .

Model	Classes	Accuracy (%)	Prediction dataset			Params (M)	FLOPs (G)
			Precision (%)	Recall (%)	F1-score (%)		
ShuffleNet_CO	Healthy Broken Mildly moldy Severely moldy	ACC_T_=99.93 ACC_V_=96.47 ACC_P_=95.95	88.89 97.80 98.13 98.92	97.78 89.00 99.06 97.87	93.12 93.19 98.59 98.40	0.957	0.119
ShuffleNet_CSP	Healthy Broken Mildly moldy Severely moldy	ACC_T_=99.74 ACC_V_=96.47 ACC_P_=96.62	87.50 98.96 100.00 100.00	98.91 87.96 100.00 100.00	92.86 93.14 100.00 100.00	0.918	0.109

## 5 Discussion

In the application of recognizing agricultural product damages, HSI has been widely used as a mainstream, rapid, and non-destructive measurement method that can provide morphological and compositional information. However, as for the crop kernel of small sizes, the low spatial resolution of HSI leads to weak recognition accuracy (Fabiyi et al., 2020). High spatial resolution is easily obtained from RGB images. In this study, SR-HSI images were generated by fusion of HSI and RGB images to identify soybean kernel damages.

**Figure 7:**
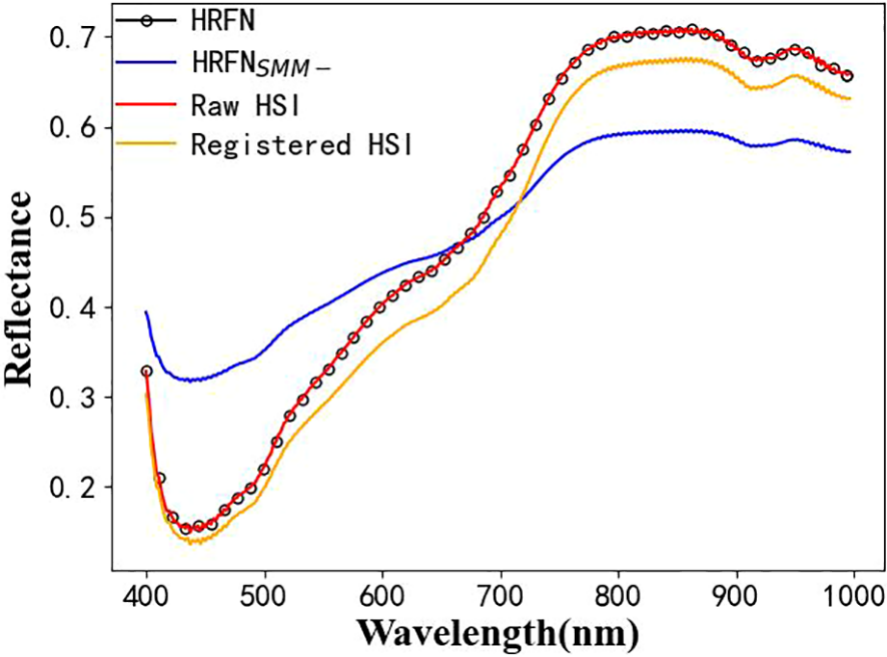
Spectral reflectance for the broken class of soybean as obtained by the raw HSI, registered HSI, and the fused results of HRFN and HRFN_SMM-_.

In most image fusion studies based on public datasets, the images from different sources are pre-registered; however, the HSI and RGB image pairs of kernels have the obvious nonlinear appearance differences in our experiments (Zhang et al., 2021). Perspective deformation was used to register image pairs and eliminate spatial dislocation. Thus far, the image fusion methods based on deep learning networks are advantageous because the networks can extract the targeted features and achieve adaptive feature fusion. The source image set has no real GT image, so the networks of supervised learning are unapplicable. A network based on unsupervised AE architecture and dense blocks, called as SRM, was constructed to fuse image pairs from HSI and RGB. The above fusion can ensure the spatial quality of images well, but the spectral information of raw HSI is difficult to guarantee. Based on the experiment, the spectral trend of the super-resolution images obtained by SRM was distorted (**Figure 7**). Referring to the channel attention mechanism, the new branch network was developed and called SMM to extract spectral details by learning the mapping relationship between the HSI and super-resolution images and accomplish spectral correction. By integrating SRM and SMM, HRFN achieved a good fusion of HSI and RGB images, that is, the FMI_pixel, MS_SSIM, and Qabf of the four classes of soybean kernels were 0.9415–0.9614, 0.9678–0.9880, and below 0.0508. The SR-HSI image with high spatial and excellent spectral resolution are expected to provide more accurate results for analysis of soybean damages.

An SR-HSI image of soybean kernels contains images of 272 wavelengths with redundant information, resulting in low processing efficiency and huge modeling cost. Thus, selecting the SR-HSI image of many significant wavelengths is essential to damage identification (Weng et al., 2021). Here, candidate EWs were first selected by SVM models developed with spectral reflectance of each wavelength, and OISEW was finalized by ShuffleNet and the successive superposition of monochromatic images of each EW. The ACC_P_=92.27%, Params=1.258 M, and FLOPs=0.155 G of ShuffleNet with OISEW are insufficient to identify damaged kernels in the real world within the limited computational budget. ShuffleNet_COCSP was constructed by adding the CO operation and the CSP architecture into ShuffleNet and obtained ACCp=98.36%, Params=0.805 M, and FLOPs=0.097 G, outperforming ShuffleNet, MobileNetV2, and GhostNet. The increase in the depth and kernel size of convolution extended the effective receptive field and led to enhanced promotion of the networks (Luo et al., 2016). However, the former brings optimization problems. Thus, the CO operation increased the kernel size from 3 × 3 to 7 × 7 and removed the last convolution of 1 × 1 to increase the perceived area of the feature map and extract rich global features. Meanwhile, shortcut is especially vital for networks with large convolution kernels (Ding et al., 2022). CSP with ingenious shortcut operation was induced to reduce the possibility of duplication in information integration and alleviate oversmoothing (Wang et al., 2020), thereby improving the learning ability of the network.

Based on the ablation experiments, the combination of the CO operation and the CSP architecture is better than the single optimization. That is, ShuffleNet_COCSP had ACCp that increased by 2.41% and 1.74%, parameters that decreased by 15.88% and 12.31%, and FLOP that decreased by 18.49% and 11.01% compared with ShuffleNet_CO and ShuffleNet_CSP, respectively. Further, ShuffleNet_COCSP and HSI and RGB images were used to identify soybean kernel damages (**Table 6**). The ACCp=95.82% of HSI and the ACCp=96.12% of RGB were worse than those of SR-HSI. The SR-HSI images are more discriminative than the HSI images in the subtle information of external features, such as texture and edge, and have wider wavelength perception and stronger diffraction ability than RGB to better identify the internal tissue characteristics of soybean kernels (Sharma et al., 2016).

**Table 6:** Classification results of ShuffleNet_COCSP based on HSI and RGB images.

Data	Classes	Accuracy (%)	Prediction dataset		
			Precision (%)	Recall (%)	F1-score (%)
HSI	Healthy Broken Mildly moldy Severely moldy	ACC_T_= 99.99 ACC_V_= 96.71 ACC_P_= 95.82	90.43 93.40 100.00 98.97	92.39 91.67 99.11 100.00	0.91.40 0.92.52 0.99.55 0.99.48
RGB	Healthy Broken Mildly moldy Severely moldy	ACC_T_=100.00 ACC_V_= 97.64 ACC_P_= 96.12	100.00 98.18 92.24 94.90	91.30 100.00 95.54 96.88	95.45 99.08 93.86 95.88

The damaged soybean kernels identified were accurately analyzed by fusion of HSI and RGB and ShuffleNet_COCSP. However, some aspects need further optimization to obtain better application prospects. The acquisition method was unpractical owning to source images from HSI and RGB cameras in our work. In the future, customized and simplified imaging equipment should be developed to easily obtain EW and RGB images. ShuffleNet_COCSP with small network size and fast recognition speed will be embedded in mobile devices to provide a wide range of application scenarios for intelligent soybean sorting. In contrast to the orderly arrangement of soybeans in this experiment, soybeans in actual sorting equipment have overlapping and adhesion phenomenon. Therefore, image segmentation and image correction in complex background should be considered.

## 6 Conclusion

In this work, damages to soybean kernels were identified using the improved ShuffleNet and fusion of HSI and RGB. The HSI and RGB image pairs of healthy, broken, and moldy soybean kernels were collected and registered by perspective deformation to eliminate spatial misalignment. HRFN, an unsupervised fusion network, was designed using SRM and SMM in parallel to generate SR-HSI with high spatial resolution and excellent spectral resolution. HRFN achieves a good fusion of HSI and RGB images for the four classes of soybean kernels, with FMI_pixel of 0.9415–0.9614, MS_SSIM of 0.9678–0.9880, Qabf below 0.0508, and perfectly preserved spectral information. Six EWs were selected by SVM, and the OISEW composed of the monochromatic images in 771, 491, 700, 927, and 635 nm was further screened by ShuffleNet. ShuffleNet_COCSP was constructed by adding the CO operation and the CSP architecture into ShuffleNet, and the best result was obtained with ACCp=98.36%, Params=0.805 M, and FLOPs=0.097 G, outperforming MobileNetV2, GhostNet, and the cases of HSI and RGB images. The high-quality SR-HSI images obtained by fusing HSI and RGB images can quickly and accurately identify small kernels, and a customized simplified imaging device can be designed to acquire SR-HSI images with scattered wavelength to meet the practical requirement of damaged kernel identification in the future. The lightweight ShuffleNet_COCSP will be deployed in mobile devices for large-scale detection of damaged kernels and real-time management in the future. In addition, advanced image correction is indispensable due to environmental factors, such as position of imaging devices and motion of samples, causing kernels to overlap one another in the sorting equipment.

## Data availability statement

The original contributions presented in the study are included in the article/**Supplementary Material**, further inquiries can be directed to the corresponding author/s.

## Author contributions

LZ: Conceptualization, Methodology, Validation, Funding acquisition, Writing – review & editing. MZ: Conceptualization, Methodology, Writing original draft, Software, Datacuration, Writing – review & editing. JZhu: Conceptualization, review & editing. LH: Visualization, Investigation. JZhao: Supervision. DL: Supervision. DZ: Supervision. All authors contributed to the article and approved the submitted version.
