# Supplementary material for: Fusion of hyperspectral imaging (HSI) and RGB for identification of soybean kernel damages using ShuffleNet with convolutional optimization and cross stage partial architecture
Source: Front Plant Sci. 2023 Jan 18;13:1098864. doi: 10.3389/fpls.2022.1098864 (PMC9889993; doi:10.3389/fpls.2022.1098864)
Supplement: Supplementary file 1 [file DataSheet_1.pdf]

Fusion of hyperspectral imaging (HSI) and RGB for identification of soybean kernel damages using ShuffleNet with convolutional optimization and cross stage partial architecture

Ling Zheng[a], Mingyue Zhao[a], Jinchen Zhu[a], Linsheng Huanga[a,*], Jinling Zhao[a],

Dong Liang[a], Dongyan Zhang[a]

National Engineering Research Center for Agro-Ecological Big Data Analysis & Application, Anhui University, 111 Jiulong Road Hefei, China

*Corresponding authors:

Professor, Linsheng Huang, Anhui University, Hefei 230601, Anhui, People's Republic of China.

Supplementary Material

Four kinds of soybean kernels

Healthy soybean kernels, broken soybean kernels, mildly moldy and severely moldy soybean kernels (**Fig. S1**) were obtained from agricultural Management Company in Shu County, China.

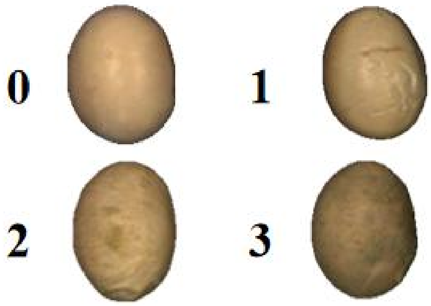

Fig. S1. Images of soybean kernels of different damages; Class 0–3: healthy, broken, mildly moldy and severely moldy soybean kernels.

RGB and hyperspectral imaging system

RGB and hyperspectral imaging system (**Fig. S2**) is comprised of a hyperspectral imager (Headwall Photonics Inc., Bolton, MA, USA), a industrial camera (HIKVISION MV-CA060-11GM),two halogen neodymium lamps (75 W) and a computing unit.

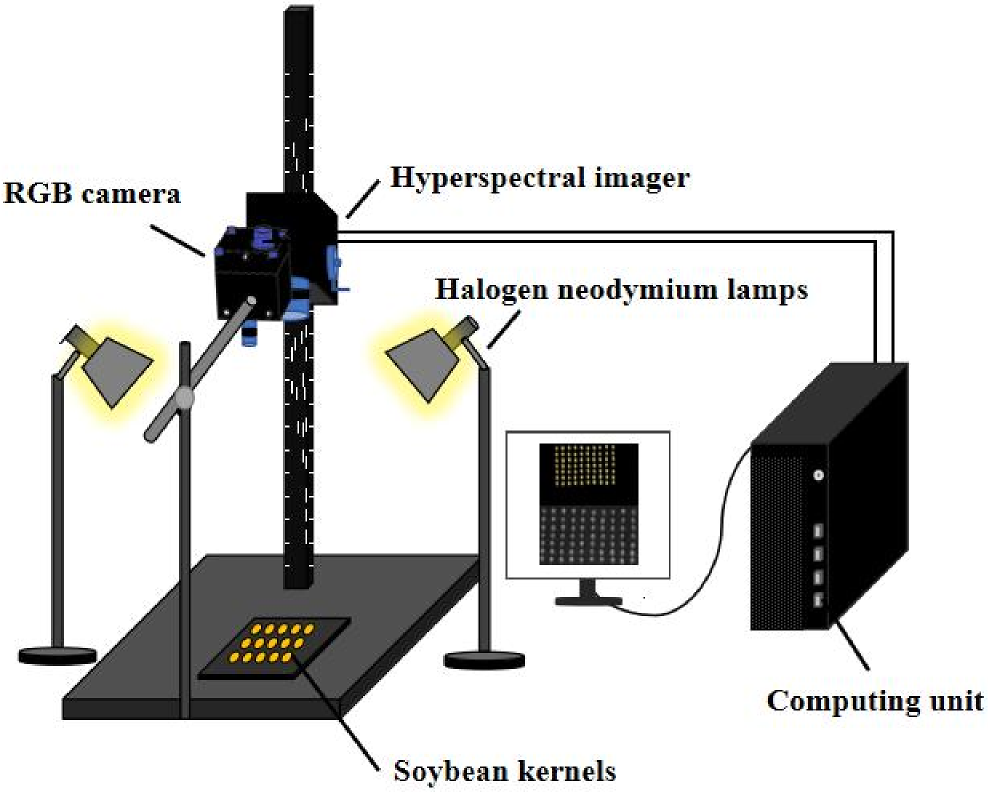

Fig. S2. Schematic diagram of RGB and hyperspectral imaging system.

Parameter setting of models

The parameter settings for the SVM, HRFN, MobileNetV2, GhostNe, ShuffleNet and ShuffleNet_COCSP models are shown in **Table S1**.

Table S1. Parameter setting of different classification models.

Methods	Parameters
**SVM**	Kernel function: 'RBF', cost=5, gamma=20, degree=3, decision function: 'ovr', class_weight: 'balanced'
**HRFN**	Encoder(Relu): Conv2d_1@3 × 3; stride=1; Channel(input)=1, Channel(output)=16;<br>Dense(dense block) (Relu) Conv2d_2@3 × 3; stride=1; Channel(input)=16, Channel(output)=16; Conv2d_3@3 × 3; stride=1; Channel(input)=32, Channel(output)=16; Conv2d_4@3 × 3; stride=1; Channel(input)=48, Channel(output)=16;<br>Decoder (Relu) : Conv2d_5@3 × 3; stride=1; Channel(input)=64, Channel(output)=64; Conv2d_6@3 × 3; stride=1; Channel(input)=64, Channel(output)=32; Conv2d_7@3 × 3; stride=1; Channel(input)=32, Channel(output)=16; Conv2d_8@3 × 3; stride=1; Channel(input)=16, Channel(output)=1;<br>optimizer: 'Adam', loss: mse_loss+ ssim_loss, batch_size=2, epochs=4, learning rate= 0.0001

**MobileNetV2**	Convolution_1 (Relu) input:224;Conv2d_1@3 × 3, stride=2<br>Bottleneck (Relu) 112;Conv2d_2@1 × 1, DWConv 3 × 3, Conv2d_3@1 × 1, Add (Conv2d_2, Conv2d_2), stride=1<br>Bottleneck (Relu) 112; Conv2d_4@1 × 1, DWConv 3 × 3, Conv2d_5@1 × 1, stride=2, Repeat 2<br>Bottleneck (Relu) 56; Conv2d_6@1 × 1, DWConv 3 × 3, Conv2d_7@1 × 1, stride=2, Repeat 3<br>Bottleneck (Relu) 28; Conv2d_8@1 × 1, DWConv 3 × 3, Conv2d_9@1 × 1, stride=2, Repeat 4<br>Bottleneck (Relu) 14; Conv2d_10@1 × 1, DWConv 3 × 3, Conv2d_11@1 × 1, Add (Conv2d_2, Conv2d_2), stride=1, Repeat 3<br>Bottleneck (Relu) 14;Conv2d_12@1 × 1, DWConv 3 × 3, Conv2d_13@1 × 1, stride=2, Repeat 3<br>Bottleneck (Relu) 7;Conv2d_14@1 × 1, DWConv 3 × 3, Conv2d_15@1 × 1, Add (Conv2d_14, Conv2d_15), stride=1,<br>Globalaveragepooling_1;<br>Convolution_2 (Relu);Conv2d_16@1 × 1<br>Fully Connected_1 (Relu) 4<br>optimizer: 'Adam', loss: 'categorical_crossentropy', batch_size=20, epochs=400, learning rate= 0.0001
**GhostNet**	Convolution_1 (Relu) input:224;Conv2d_1@3 × 3, stride=2<br>GhostBottleneck (Relu) 112;Conv2d_2@3 × 3, stride=1<br>GhostBottleneck (Relu) 112;DWConv 3× 3, stride=2<br>GhostBottleneck (Relu) 56;Conv2d_3@3 × 3, stride=1<br>GhostBottleneck (Relu) 56;DWConv 3× 3, stride=2<br>GhostBottleneck (Relu) 28;Conv2d_4@3 × 3, stride=1<br>GhostBottleneck (Relu) 28;DWConv 3× 3, stride=2<br>GhostModule (Relu)14;Conv2d_5@1 × 1, stride=1,last stride=2<br>GhostModule (Relu)7;Conv2d_6@1 × 1, stride=1<br>Globalaveragepooling_1;<br>Convolution_2 (Relu);Conv2d_7@1 × 1, stride=1<br>Fully Connected_1 (Relu) 4<br>optimizer: 'Adam', loss: 'categorical_crossentropy', batch_size=20, epochs=400, learning rate= 0.0001
**ShuffleNet**	Convolution_1 (Relu) 224;Conv2d_1@3 × 3; Max-pooling_1@3 × 3<br>Stage1 (Relu) 56; Downsampling unit (Relu); path1: Conv2d_2@1 × 1, DWConv 3 × 3, Conv2d_3@1 × 1, Path2: DWConv 3 × 3, Conv2d_4@1 × 1, stride=2, Basic unit (Relu); Conv2d_5@1 × 1, DWConv 3 × 3, Conv2d_6@1 × 1, stride=1, Repeat 3<br>Stage2 (Relu) 28; Downsampling unit (Relu); path1: Conv2d_7@1 × 1, DWConv 3 × 3, Conv2d_8@1 × 1, Path2: DWConv 3 × 3, Conv2d_9@1 × 1, stride=2, Basic unit (Relu); Conv2d_10@1 × 1, DWConv 3 × 3, Conv2d_11@1 × 1, stride=1, Repeat 3<br>Stage3 (Relu) 14; Downsampling unit (Relu); path1: Conv2d_12@1 × 1, DWConv 3 × 3, Conv2d_13@1 × 1, Path2: DWConv 3 × 3, Conv2d_14@1 × 1, stride=2, Basic unit (Relu); Conv2d_15@1 × 1, DWConv 3 × 3, Conv2d_16@1 × 1, stride=1, Repeat 3

	Convolution_2 (Relu) 7;Conv2d_17@1 × 1<br>Globalaveragepooling_1;<br>Fully Connected_1 (Relu) 4<br>optimizer: 'Adam', loss: 'categorical_crossentropy', batch_size=20, epochs=400, learning rate= 0.0001
**ShuffleNet_COCSP**	Convolution_1 (Relu) 224;Conv2d_1@3 × 3; Max-pooling_1@3 × 3<br>Stage1 (Relu) 56; csp_conv (Relu), Downsampling (Relu); path1:DWConv 7 × 7, Conv2d_2@1 × 1, Path2: Conv2d_3@1 × 1, DWConv 7 × 7, stride=2, Basic (Relu); Conv2d_4@1 × 1, DWConv 7 × 7, stride=1, Repeat 3, Add (csp_conv, Basic)<br>Stage2 (Relu) 28; csp_conv (Relu), Downsampling (Relu); path1:DWConv 7 × 7, Conv2d_5@1 × 1, Path2: Conv2d_6@1 × 1, DWConv 7 × 7, stride=2,Basic (Relu); Conv2d_7@1 × 1, DWConv 7 × 7, stride=1, Repeat 3,Add (csp_conv, Basic)<br>Stage3 (Relu) 14; csp_conv (Relu), Downsampling (Relu); path1:DWConv 7 × 7, Conv2d_8@1 × 1, Path2: Conv2d_9@1 × 1, DWConv 7 × 7, stride=2,Basic (Relu); Conv2d_10@1 × 1, DWConv 7 × 7, stride=1, Repeat 3,Add (csp_conv, Basic)<br>Convolution_2 (Relu) 7;Conv2d_11@1 × 1<br>Globalaveragepooling_1;<br>Fully Connected_1 (Relu) 4<br>optimizer: 'Adam', loss: 'categorical_crossentropy', batch_size=20, epochs=400, learning rate= 0.0001

batch_size — the number of training samples sent into the network for each training.

epochs — total number of training sessions for all samples.
